# Effect of computerized cognitive training on mood, cognition, and serum brain-derived neurotrophic factor level in late-life depression — a pilot randomized controlled trial

**DOI:** 10.3389/fpsyt.2023.1287822

**Published:** 2024-01-17

**Authors:** Suk Ling Ma, Katsie Tung Tang, Niki Cheuk Ting Lau, Clement Lek Hin Chiu, Cuichan Lin, Linda Chiu Wa Lam, Allen Ting Chun Lee

**Affiliations:** Department of Psychiatry, Faculty of Medicine, The Chinese University of Hong Kong, Shatin, Hong Kong SAR, China

**Keywords:** computerized cognitive training (CCT), mood, cognition, brain-derived neurotrophic factor (BDNF), depression, randomized controlled trial

## Abstract

**Background:**

The aim of this pilot randomized controlled trial was to test the feasibility of a computerized cognitive training targeting executive dysfunction in late-life depression and to investigate its impact on mood, cognition, and brain-derived neurotrophic factor (BDNF) levels.

**Methods:**

A total of 28 community-living Chinese individuals aged 55–75 with moderate-to-severe depression and cognitive symptoms (but without mild cognitive impairment or dementia) were recruited from a community centre in Hong Kong. Participants were randomly allocated to either the experimental (receiving computerized cognitive training) or the control group (receiving computer-based health education). Both programs lasted for one hour and were conducted twice a week for 6 weeks at the community centre. We assessed mood using the Hamilton Rating Scale for Depression (HAM-D) and Patient Health Questionaire-9 (PHQ-9), cognition using the Montreal Cognitive Assessment (MoCA), and serum BDNF levels at baseline and follow-up. We performed repeated measures analysis of variance to compare the differences in outcome changes between groups and correlation analysis to test if changes in mood and cognition correlated with changes in BDNF level.

**Results:**

Our sample had a mean age of 66.8 (SD = 5.3) years, a mean HAM-D score of 19.4 (SD = 7.5), and a mean PHQ-9 score of 18.0 (SD = 6.3). No adverse effects were reported. Significant differences were observed between the experimental and control groups in changes in HAM-D (-8.4 vs. -2.9; group difference = -5.5; *p* = 0.01), PHQ-9 (-6.6 vs. -0.6; -6.0; *p* < 0.001), MoCA (1.4 vs. -1.3; 2.7; *p* = 0.001), and serum BDNF levels (in pg/ml; 2088.3 vs. -3277.4; 5365.6; *p* = 0.02). Additionally, changes in HAM-D, PHQ-9, and MoCA scores correlated significantly with changes in BDNF level.

**Conclusion:**

With computerized cognitive training improving mood and cognition and increasing serum BDNF levels in 6 weeks, it may serve as a safe and effective evidence-based alternative or adjuvant treatment for late-life depression.

**Clinical trial registration:**

https://www.chictr.org.cn/indexEN.html, identifier ChiCTR1900027029.

## Introduction

Late-life depression (LLD) is prevalent among older people (1, 2). Not only does it contribute to a high elderly suicide rate, which is twice as high as the adult population, but it also doubles the risk of dementia (2, 3). Yet, treating LLD is more challenging than adult depression – less than half respond to drug treatment; only one-third achieve remission, half of which recur within 2 years; and older adults are more prone to side-effects (4, 5). Notably, executive dysfunction, a common feature in LLD, predicts poor treatment response and often persists after mood symptoms remit (6). Developing an alternative or adjuvant treatment for LLD is therefore of great clinical and public health importance, especially considering our rapidly ageing populations.

Given the benefits of computerized cognitive training (CCT) in slowing cognitive decline among older adults with neurocognitive disorders and its cognitively stimulating effects among cognitively unimpaired older adults (7–9), CCT that targets frontal lobe and improves executive function might be a promising treatment for LLD (10, 11). Compared to existing interventions, CCT is standardized, structured, repeatable, more accessible and cost-effective than psychotherapy, allows monitoring of adherence, can be tailored to individual skill level as a form of personalized medicine, and does not have concerns for drug side-effect or drug–drug interaction (12, 13). Although numerous CCT apps are available in the market, few have undergone vigorous randomized controlled trials (RCTs) and demonstrated evidence-based clinical validity in LLD (13). The amount of exposure to CCT also varies across studies, though current practice often requires 30–60 min/session over 2–10 weeks (i.e., ~10 h or 500 min of training) for an appreciable effect to be seen (11), with effects plateauing off or diminishing thereafter due to cognitive fatigue from overtraining (13). More importantly, the neurobiological mechanisms of CCT improving mood and cognition have yet to be delineated. A recent RCT showed that CCT improves cognition and increases serum level of brain-derived neurotrophic factor (BDNF) – a neurotrophic factor important for neurogenesis and synaptic plasticity and implicated in depression and cognitive impairment (14, 15) – in non-depressed older adults (16, 17); nevertheless, evidence of CCT improving LLD via BDNF is lacking. Trials that can unveil the mechanisms underlying mood and cognitive improvement are crucial in demonstrating that CCT does not simply “teach to test” and that it can serve as a promising evidence-based treatment for LLD.

Our team designed a structured frontal lobe-based CCT and conducted a small pilot RCT in a community centre, aiming to test the feasibility and, importantly, the effect of CCT in older adults with clinical depression and cognitive symptoms [but without mild cognitive impairment (MCI) or dementia]. The primary objective was to identify the effect of CCT on mood symptoms in LLD. The secondary objectives were to identify the effect of CCT on cognitive function and serum BDNF levels, and the correlations between changes in mood and cognition and changes in BDNF levels. The working hypotheses were that CCT improved mood symptoms, cognitive function, and serum BDNF levels, and that the improvement observed in mood and cognition correlated with increased BDNF levels. The anticipatory evidence generated from this RCT would shed light on the potential of CCT in helping older adults overcome LLD and the biological mechanisms of CCT in improving LLD.

## Materials and methods

### Study design

This pilot study was a two-arm parallel-design RCT conducted in Hong Kong from 25 October 2019 to 29 June 2022, with enrollment from 24 November 2020 to 18 November 2021. The framework of this trial was to test the feasibility and superiority of CCT over a health education program, primarily focusing on mood outcomes in LLD. This study was registered with the Chinese Clinical Trial Registry (reference number: ChiCTR1900027029) and was approved by and conducted in accordance with the ethical standards of the Joint Clinical Research Ethics Committee of the Chinese University of Hong Kong and the New Territories East Cluster of the Hospital Authority (reference number: CREC-2019.497) and the Declaration of Helsinki. Written consent was obtained from all subjects before recruitment.

### Participants

Individuals attending our collaborating community centre were recruited by centre staff and screened by our trained research staff for eligibility to participate. Inclusion criteria were aged 55–75 years, ethnic Chinese, living in the community, having a primary diagnosis of major depressive disorder (single or recurrent episodes without psychotic features) based on the DSM-5 criteria (18), with moderate-to-severe depression (scoring ≥17 in the Hong Kong Chinese version of the 17-item Hamilton Rating Scale for Depression; HAM-D) and cognitive symptoms [scoring ≥2 in Item 8 (slowness of thought and speech, impaired ability to concentrate) of HAM-D] (19), but without MCI or dementia (based on the age- and education-specific cutoff of the Hong Kong Chinese version of the Montreal Cognitive Assessment; MoCA) (20). Individuals already attending psychiatric clinic or receiving antidepressant were included, provided that they were not expected to start or had already been on the same regimen for the past 6 months; this helped to ensure that the observed response was attributed to the CCT, rather than being an effect of a new dosage of antidepressants or the introduction of a new medication. However, those who were treatment-resistant (history of electroconvulsive therapy, transcranial magnetic stimulation, or no clinical response to ≥2 different classes of antidepressants) were excluded, and those with active suicidal idea or psychotic symptom were referred to psychiatric services for further management. Other exclusion criteria were non-Chinese; living in care homes; having history or ongoing alcohol or substance abuse, mania, bipolar or psychotic disorder, post-traumatic stress disorder, anxiety disorder, personality disorder, or mental disorder due to organic cause; having history of traumatic brain injury, Parkinson’s disease, stroke, MCI, dementia, or scoring below MoCA cutoff; taking drugs that affect cognition (such as benzodiazepines, cholinesterase inhibitors, memantine, and anticholinergics); attending other cognitive or psychological intervention in the past 6 months; refusing blood taking; or having language barrier or communication difficulties (poor vision or hearing despite correction). A total of 28 participants were recruited into this study.

### Randomization and blinding

Recruited subjects were randomly allocated in a 1:1 ratio to either the experimental (CCT) or control (health education) group. The randomization list was generated by a research staff who was not involved in enrolment or assessment, using a computer.^1^ Participants were assumed blinded as both groups received computer-based intervention of the same frequency, duration, and length in different rooms. Moreover, they were reminded not to discuss their intervention with other participants or outcome assessor. Furthermore, to reduce expectancy bias, participants were explained at the time of seeking consent that there was no prior evidence to support a difference in benefit between the two groups. The research staff who assisted in consent seeking and intervention were not involved in outcome assessment, and those who assessed outcomes were blinded to the allocation status without involvement in intervention.

### Intervention

#### Experimental group (computerized cognitive training)

Our structured CCT was specifically designed to target the cognitive domains involved in LLD, namely (1) complex attention, (2) executive control, and (3) working memory. The structure of the CCT in each session and details of training on each cognitive domain are described in the Supplementary information. Each session consisted of 4 training tasks, each lasting for 10–15 min, with a short break in between. All participants began the training with a trial run, followed by varying blocks of trials depending on the cognitive domain being trained. Upon completion of each trial and before moving onto the next one, the score would be revealed to the participant to provide instant feedback.

#### Control group (health education)

Participants randomized to the control group attended a computer-based health education program, which consisted of a series of health videos on common health problems in older adults, aiming to improve their general health. The program had the same length, frequency, duration, and mode of delivery as our CCT, but without significant frontal lobe activation.

### Procedure

Both groups underwent one hour of intervention twice a week for 6 weeks at the community centre. Interventions were conducted in small groups (4 participants/group) in spacious, quiet activity rooms at our collaborating community centre. The experimental and control groups received interventions separately to prevent interaction. We specifically chose not to adopt online CCT as less than half of the older adults in our locality have access to a tablet or computer at home (21). Although 70% of older adults have smartphones (21), the screen size is too small for 1-h training. Moreover, based on our past experience, intensive and close staff support was needed to assist or promote regular online training. As local older adults enjoy attending training in person, we conducted our intervention at the community centre.

Each participant received the intervention at their own station, equipped with an individual table, chair, and tablet computer pre-installed with our CCT and connected with a large USB number pad (which facilitated older adults to press the buttons). Our trained research staff set up the stations on site, explained the instructions to participants before each training task, and supervised them throughout the session. The staff was instructed not to assist participants in identifying the correct answers during training. Each participant was given a unique user ID to log into our computer program, allowing attendance and scores to be recorded and ensuring that the control group could not receive the CCT (and vice versa). To minimize distraction and the confounding effect of social engagement, participants were advised not to communicate with others during the session. To ensure training consistency, each group had a set time of training, and the same research assistant was assigned to conduct all the sessions.

To promote adherence and minimize dropout, we adopted the following measures: (1) Prior to collecting written informed consent, our research staff explained our program in detail to eligible individuals (aims, number of sessions, requirements, etc.). An information sheet was also provided, so that individuals had a clear idea of the expected commitment before deciding to participate. (2) Our program was free of charge. (3) We designed the training with an aim to maintain participants’ interest but without gaming components. (4) Our research staff reminded participants by phone before each session. (5) A dedicated hotline was set up to address participants’ queries.

### Assessment

The pre-specified outcomes were mood, cognition, and serum BDNF level, which were assessed at baseline (T0; prior to randomization) and at Week 6 (T1; post-intervention).

#### Mood

The severity of depression was primarily assessed by the 17-item HAM-D, a locally validated and widely used tool for rating depression (19). The Patient Health Questionnaire-9 (PHQ-9), another locally validated tool, was used as a secondary mood outcome (22). In both assessments, a higher score indicates greater severity of depression. In HAM-D, scores of 8–16, 17–23, and ≥ 24 are regarded as mild, moderate, and severe depression, respectively (23). In PHQ-9, scores of 5–9, 10–14, 15–19, and 20–27 are regarded as mild, moderate, moderately severe, and severe depression, respectively (22).

#### Cognition

Global cognition was primarily assessed by the MoCA, a locally validated cognitive screening test sensitive in detecting early memory and executive deficits (20). The total score of MoCA is 30, with higher scores indicating better cognitive function. To increase the sensitivity of identifying cognitive complaints, we included the Memory Inventory for Chinese (MIC), a locally validated screening tool for Subjective Cognitive Decline (SCD) (24). MIC contains 27 items, with higher scores indicating more subjective cognitive complaints.

#### BDNF

Venous blood samples were collected in anticoagulant-free tubes in the morning to minimize diurnal variation in BDNF level. These were kept at room temperature for 1 h to allow clotting, and centrifuged at 3500 g for 10 min at 4°C within 3 h of blood collection to obtain serum. Aliquots were stored at -70°C until assay. A commercial ELISA kit was used to assay BDNF level, with measurement performed as per the manufacturer’s instructions. The standard curve and 20% of samples were analyzed in triplicate.

#### Covariates

Sociodemographic factors such as age, sex, years of education, and socioeconomic status (as defined by receiving comprehensive social security assistance from the government) were assessed at baseline to identify any differences.

#### Adherence rate

Adherence rate was measured by dividing the number of sessions attended by participants over the total number of sessions (i.e., 12 sessions).

#### Adverse events and unbinding

Adverse events (AEs) were expected to be rare, as our intervention did not involve any risky procedures. Nevertheless, potential risks such as mild discomfort from intense concentration on CCT were explained to participants before signing consent. Participants were advised to report any health problems or suspected AEs during the intervention period to the research team. In follow-up assessments, our research staff also screened for and documented any AEs. All complaints were reviewed by the principal investigator. The intervention would be halted immediately if the reported event was related.

### Sample size calculation

The sample size (alpha 0.05, power 0.8) required to detect significant differences in outcome changes was estimated using G*Power. Our previous meta-analysis found CCT having a medium effect size (*d* = 0.54) in reducing depressive symptoms among older adults with dementia (25). Since participants in this study were free of dementia, and our CCT was specifically designed to target frontal lobe dysfunction in LLD, the effect size of CCT in this study was estimated to be greater. Assuming an attrition rate of 10%, and given that this was a pilot RCT which primarily aimed to test the feasibility and effect of CCT on mood symptoms in LLD, a sample size of 28 (14 in each arm) would be able to detect a statistically significant mean difference in mood changes between groups.

### Statistical analyses

Statistical analysis was performed using IBM SPSS Statistics (version 27). Descriptive statistics were used to report the baseline characteristics of our participants. Using the intention-to-treat principle, repeated measures analysis of variance was used to evaluate the outcome changes in 6 weeks, with time as the within-group factor, and experimental/control as the between-group measure. Bonferroni adjustment was adopted for multiple comparisons. The above analyses were repeated in the completers-only population as post-hoc sensitivity analysis. Correlation analysis was performed to test if changes in mood and cognitive outcomes correlated with changes in BDNF level. The adherence rate, which helps reflect the feasibility of our CCT, was calculated by dividing the number of sessions attended by the participant by twelve (i.e., the total number of sessions available over 6 weeks).

## Results

### Baseline characteristics

**Figure 1:**
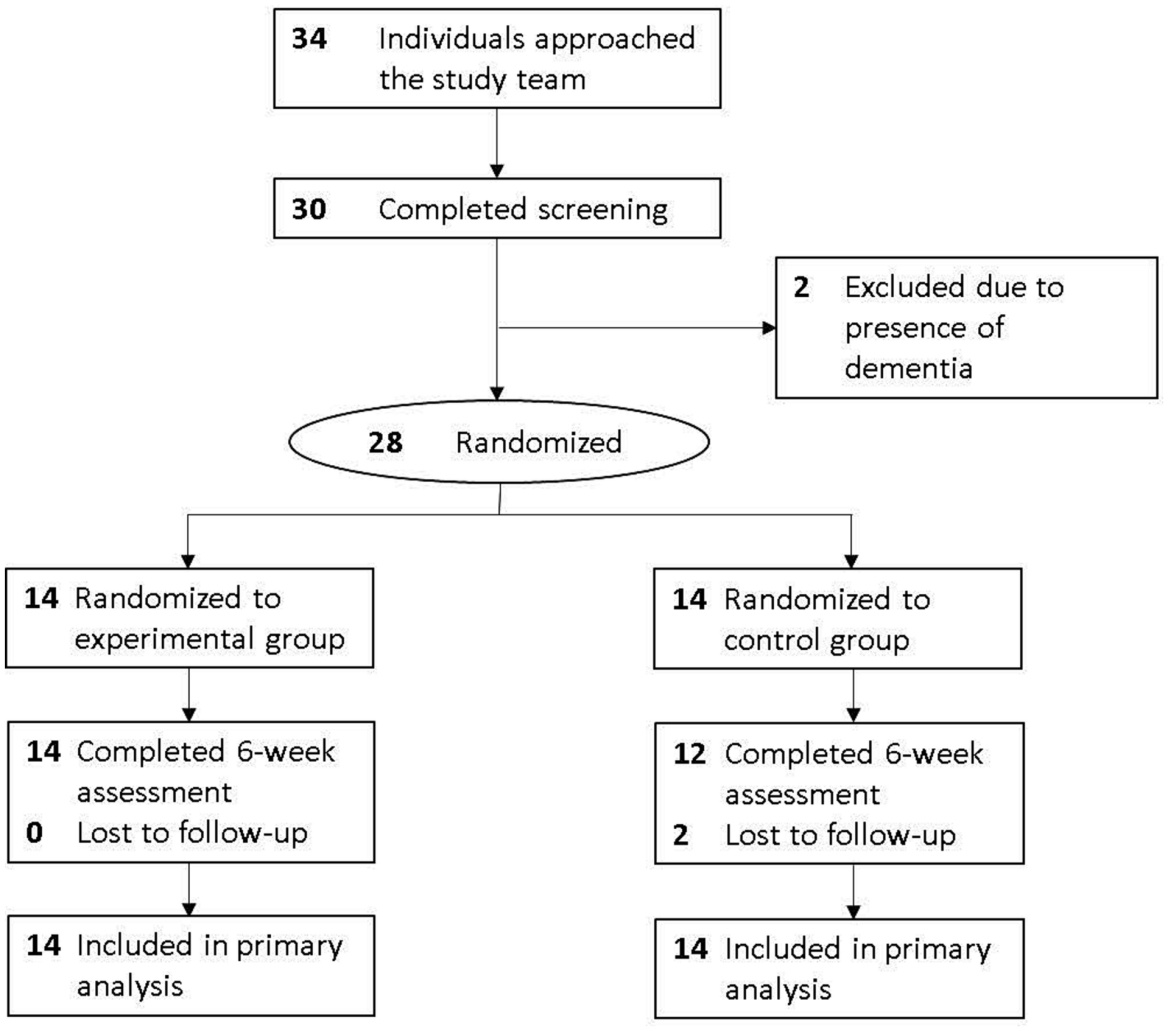
CONSORT flow diagram of this study.

A total of 34 individuals approached us, with 30 completing the screening. Of these, two did not meet the study criteria as they had dementia. Consequently, 28 subjects provided written informed consent and completed baseline assessment (Figure 1). Of these, two (7.1%) dropped out, and 26 (92.9%) completed follow-up assessment.

Our sample had a mean age of 66.8 (5.3) years, with 22 (78.6%) females, 10.4 (4.4) years of education, and 16 (57.1%) on antidepressant treatment. The mean HAM-D and PHQ-9 scores were 19.4 (7.5) and 18.0 (6.3), and the mean MoCA and MIC scores were 27.3 (1.5) and 7.1 (4.1), respectively, at baseline. Their mean serum BDNF level was 5889.8 (5925.3) pg/ml (see Table 1).

**Table 1:** Baseline characteristics of our sample.

Baseline variables, mean ± SD/number (%)	Experimental group (*n* = 14)	Control group (*n* = 14)	Whole sample (*N* = 28)
Age (years)	65.9 ± 3.5	67.7 ± 6.8	66.8 ± 5.3
Female	11 (79)	11 (79)	22 (79)
Education (years)	9.9 ± 4.0	10.8 ± 4.9	10.4 ± 4.4
Current use of antidepressant	8 (57)	8 (57)	16 (57)
HAM-D	19.3 ± 8.2	19.5 ± 6.9	19.4 ± 7.5
PHQ-9	17.4 ± 6.4	18.6 ± 6.5	18.0 ± 6.3
MoCA	26.7 ± 1.5	27.9 ± 1.4	27.3 ± 1.5
MIC	8.7 ± 2.3	5.3 ± 5.0	7.1 ± 4.1
BDNF (pg/ml)	6189.6 ± 7681.9	5556.6 ± 3517.3	5889.8 ± 5925.3

HAM-D, Hamilton Rating Scale for Depression; PHQ-9, Patient Health Questionnaire-9; MoCA, Montreal Cognitive Assessment; MIC, Memory Inventory for Chinese; BDNF, serum Brain-Derived Neurotrophic Factor level.

### Mood outcomes

**Figure 2:**
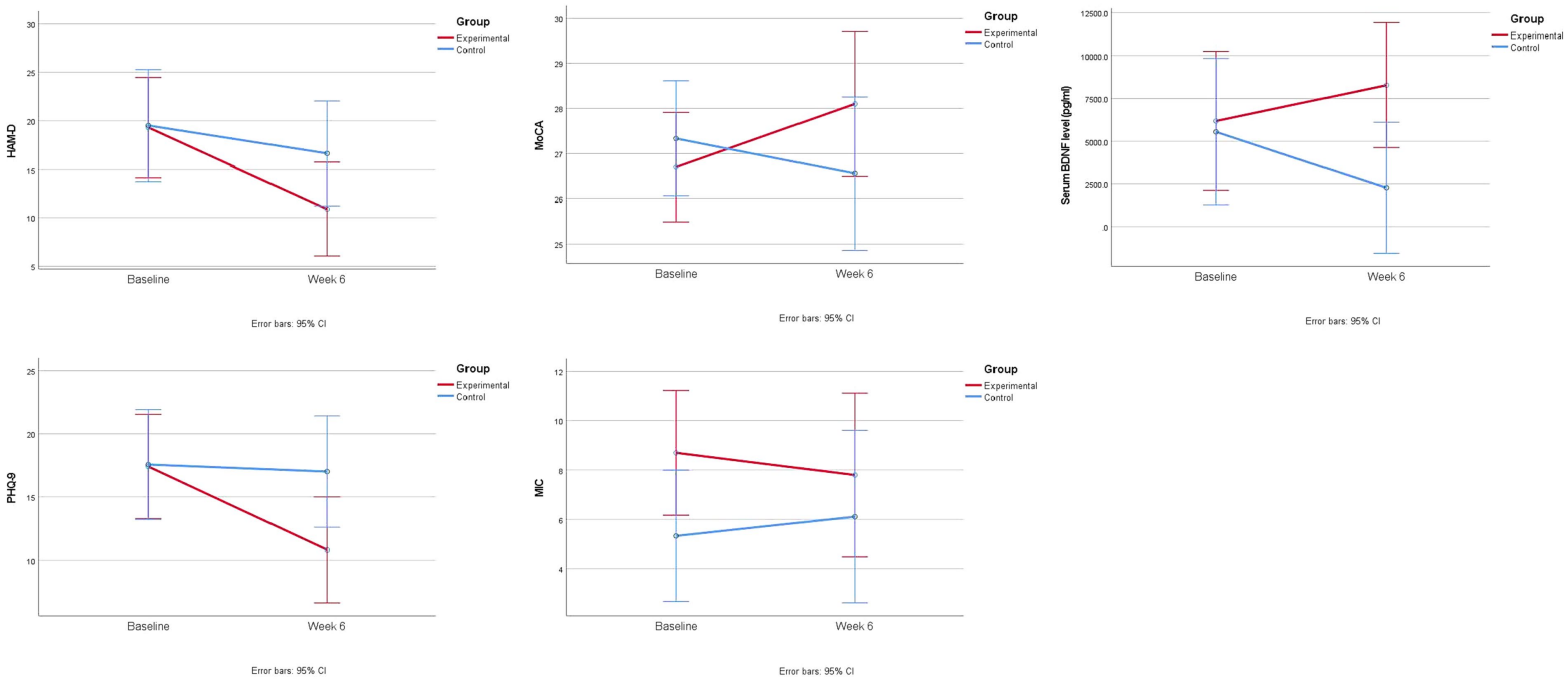
Changes in mood, cognition, and serum BDNF level over 6 weeks.

Both groups showed a statistically significant decrease in HAM-D total score over 6 weeks (mean change in experimental group = -8.4 [95% CI = -11.9 – -4.9], *p* < 0.001; mean change in control group = -2.9 [95% CI = -5.6 – -0.1], *p* = 0.04), but changes in HAM-D total score were significantly greater in the experimental group than in the control group (group difference = -5.5 [95% CI = -9.8 – -1.3], *p* = 0.01) (Table 2; Figure 2).

**Table 2:** Comparison of 6-week changes in mood and cognitive outcomes and serum BDNF level using intention-to-treat repeated measures analysis of variance.

Outcome	Experimental group				Control group				Experimental vs Control	
	Mean (SD)		Mean change (95% CI)	*p*-value	Mean (SD)		Mean change (95% CI)	*p*-value	Mean difference (95% CI)	*p*-value
	Baseline	6 weeks			Baseline	6 weeks				
HAM-D	19.3 (8.2)	10.9 (7.5)	-8.4 (-11.9 – -4.9)	<0.001	19.5 (6.9)	16.6 (6.9)	-2.9 (-5.6 – -0.1)	0.04	-5.5 (-9.8 – -1.3)	0.01
PHQ-9	17.4 (6.4)	10.8 (6.7)	-6.6 (-9.3 – -3.9)	<0.001	17.6 (6.5)	17.0 (5.7)	-0.6 (-2.6–1.5)	0.55	-6.0 (-9.2 – -2.9)	<0.001
MoCA	26.7 (1.5)	28.1 (1.8)	1.4 (0.7–2.1)	0.001	27.9 (1.4)	26.6 (3.0)	-1.3 (-2.8–0.2)	0.07	2.7 (1.3–4.2)	0.001
MIC	8.7 (2.3)	7.8 (5.4)	-0.9 (-4.6–2.8)	0.60	5.3 (5.0)	6.1 (4.5)	0.8 (-1.4–2.9)	0.43	-1.7 (-5.8–2.5)	0.40
BDNF	6189.6 (7681.9)	8277.8 (7403.6)	2088.3 (-1820.9–5997.5)	0.26	5556.6 (3517.3)	2279.3 (1243.2)	-3277.4 (-6076.7 – -478.0)	0.03	5365.6 (813.5–9917.8)	0.02

The experimental group also had a significantly greater decrease in PHQ-9 total score over 6 weeks than the control group (group difference = -6.0 [95% CI = -9.2 – -2.9], *p* < 0.001).

### Cognitive outcomes

The experimental group showed a statistically significant improvement in MoCA over 6 weeks (mean change = 1.4 [95% CI = 0.7–2.1], *p* = 0.001), whereas the control group showed a worsening trend (mean change = -1.3 [95% CI = -2.8 – 0.2], *p* = 0.07) (Table 2; Figure 2). A significant difference in changes in MoCA score was found between the two groups in 6 weeks (group difference = 2.7 [95% CI = 1.3–4.2], *p* = 0.001).

No significant between-group difference in MIC changes was found in 6 weeks (Table 2; Figure 2).

### BDNF outcome

Changes in serum BDNF level were significantly different between the two groups (Table 2), with the experimental group showing an improving trend and the control group having a decreasing trend over 6 weeks (experimental vs. control = 2088.3 pg/mL vs. -3277.4 pg/mL; group difference = 5365.6 [95% CI = 813.5–9917.8] pg/ml; *p* = 0.02).

### Other analyses

Sensitivity analyses of mood, cognitive and BDNF outcomes in the completers-only population showed results consistent with the primary analysis (Table 3).

**Table 3:** Comparison of 6-week changes in mood and cognitive outcomes and serum BDNF level using completers-only repeated measures analysis of variance.

Outcome	Experimental group				Control group				Experimental vs control	
	Mean (SD)		Mean change (95% CI)	*p*-value	Mean (SD)		Mean change (95% CI)	*p*-value	Mean difference (95% CI)	*p*-value
	Baseline	6 weeks			Baseline	6 weeks				
HAM-D	19.3 (8.2)	10.9 (7.5)	-8.4 (-11.9 – -4.9)	<0.001	19.5 (6.9)	16.6 (6.9)	-2.9 (-5.6 – -0.1)	0.04	-5.5 (-9.6 – -1.4)	0.01
PHQ-9	17.4 (6.4)	10.8 (6.7)	-6.6 (-9.3 – -3.9)	<0.001	17.0 (6.1)	16.4 (5.8)	-0.6 (-3.0–1.7)	0.55	-6.0 (-9.4 – -2.6)	0.002
MoCA	26.7 (1.5)	28.1 (1.8)	1.4 (0.7–2.1)	0.001	27.6 (1.2)	26.1 (2.9)	-1.5 (-3.2–0.2)	0.07	2.9 (1.4–4.4)	<0.001
MIC	8.7 (2.3)	7.8 (5.4)	-0.9 (-4.6–2.8)	0.60	5.9 (5.0)	6.8 (4.3)	0.9 (-1.6–3.4)	0.43	-1.8 (-6.2–2.6)	0.41
BDNF	6189.6 (7681.9)	8277.8 (7403.6)	2088.3 (-1820.9–5997.5)	0.26	5865.6 (3627.2)	2178.6 (1289.2)	-3687.0 (-6751.0 – -623.1)	0.03	5775.3 (987.1–10563.5)	0.02

Significant correlations were found between changes in HAM-D and PHQ-9 scores and changes in BDNF level (HAM-D: *r* = -0.51, *p* = 0.04; PHQ-9: *r* = -0.49, *p* = 0.03). Changes in MoCA and MIC scores also correlated significantly with changes in BDNF level (MoCA: *r* = 0.55, *p* = 0.02; MIC: *r* = -0.61, *p* = 0.01).

### Adherence rate and adverse events

Participants in the experimental group attended an average of 10 out of 12 sessions, resulting in an attendance rate of 82.6% (SD = 24.6%). No adverse effects were reported by any of the participants.

## Discussion

In this pilot RCT, we found that CCT targeting executive dysfunction in LLD improved mood, global cognition, and serum BDNF levels over a period of 6 weeks. Importantly, changes in mood and cognition correlated moderately well with the BDNF changes. The adherence rate was high, with no significant adverse effects reported. This study demonstrated the feasibility and clinical benefits of CCT in LLD and suggested that BDNF could potentially mediate the effects of CCT on mood and cognition.

While CCTs have been reported to have some benefits on mood in older adults, many have not undergone RCTs for efficacy in treating LLD. Without a control group to account for the placebo effect, it is uncertain whether the observed improvement in mood is specific to CCT (13). One recent RCT by Morimoto et al. demonstrated CCT improving mood in older adults who failed at least one trial of antidepressant (26). Nevertheless, the underlying mechanisms have yet to be established. We addressed these limitations and knowledge gap by designing a frontal lobe-based CCT, applying it to older adults with moderate to severe depression in an RCT, and examining changes in not only mood and cognition but also BDNF. Our findings support the previous literature that CCT is useful in improving mood symptoms among older adults with depression and demonstrate preliminary scientific evidence on the neurocognitive modulatory effects of CCT.

We found that improvement in mood was significantly greater in CCT than in a computer-based health education program. While computer-based health education also helps improve mood symptoms, the control group remained moderately depressed by Week 6. In contrast, the experimental group improved from moderate to mild depression. Our findings suggest that repeated training on cognitive domains involved in LLD is helpful in alleviating mood symptoms, and CCT may serve as an alternative or adjuvant treatment for LLD.

Although our CCT was specifically designed to target executive dysfunction in LLD, we observed that the experimental group showed an improvement in global cognition. This suggests that the benefits of CCT may extend beyond its primary target area, potentially enhancing overall cognitive function. Indeed, according to a recent meta-analysis, cognitive rehabilitation improves various cognitive domains, including executive function, verbal learning, and working memory, in people with depression (27). Further research is needed to explore this promising finding in more detail and to determine whether the effects of CCT translate into improvements in daily functioning (28). Interestingly, no significant difference in MIC changes was found between the two groups. As all our participants had cognitive symptoms at baseline, and their depression was not yet in complete remission after CCT, we speculate that they might remain worried about their cognitive decline and do not readily recognize their cognitive improvement as demonstrated in the objective cognitive tests. The effect of CCT on subjective cognitive changes might be better appreciated with a longer intervention or follow-up period.

A significant finding of this study is that, in contrast to the control group which exhibited a decrease in BDNF levels over time, CCT reversed this trend, resulting in higher BDNF levels by Week 6. Importantly, significant correlations exist between changes in mood and cognitive outcomes and changes in BDNF levels. These suggest that CCT may exert a neuromodulatory effect in LLD. We speculate that our frontal lobe-based CCT stimulates dorsolateral prefrontal cortex, thereby improving stress resilience via enhancing cognitive flexibility and optimizing mood regulation via modulating neuroplasticity, both of which involve BDNF. Although we could not measure the BDNF level in our participants’ brain directly, animal studies show that brain-tissue BDNF level correlates well with serum BDNF level (29), with a substantial amount of the circulating BDNF originating from the brain (30). Previous animal models show that chronic stress reduces BDNF in the brain, while treatment increases BDNF expression, promotes synaptic plasticity in the medial prefrontal cortex, and improves cognitive function (14). Similarly, prior clinical studies report that serum BDNF levels are reduced in people with depression but increase with antidepressant treatment, and such increase is associated with not only mood and cognitive improvement but also modulation of the overactive default mode network in the brain (15). Further studies should examine the neuroimaging biomarkers and sequence of events following CCT to better understand its mechanisms.

### Strengths and limitations

Regarding the strengths of this study, we recruited clinically depressed older adults and excluded those whose cognitive impairments were caused by dementia or other disorders. Our CCT was specifically designed to target cognitive dysfunctions in LLD, and measures were taken to minimize the confounding effects of social interactions and recreational or gaming components that are often present in group-based or computer-based interventions. Additionally, we included a biological outcome (serum BDNF level) to ascertain that CCT did not simply “teach to test”.

Nevertheless, this RCT had several limitations. Firstly, as this was a pilot clinical trial, the sample size was small, and the intervention and follow-up lasted for only 6 weeks. A larger and longer trial is warranted to replicate these findings and to ascertain the long-term effect of CCT. Secondly, we only recruited older adults with moderate to severe depression. It would be interesting to see whether those with milder forms and those with severe or treatment-resistant depression experience the same benefits. Thirdly, our control group ideally should differ from the experimental group by only one parameter. However, active controls often involve different cognitive domains, and it would be nearly impossible to design one that is completely free of executive functioning. Hence, we strategically chose health education as our active control while being mindful in our design to match the other components of CCT. Lastly, BDNF expression varies with different factors. In particular, response to antidepressants is lower in patients with Met allele of BDNF Val66Met polymorphism (31). Establishing the effect of this polymorphism on CCT-induced BDNF changes could help screen for individuals who are more likely to respond to CCT. Similarly, physical exercise is known to affect serum BDNF levels (32). Future research exploring the effect of CCT on BDNF levels should therefore take into account the potential influence of physical exercise on this association.

Looking ahead, the potential impact of CCT on older adults with LLD could be substantial. If further research continues to support these findings, CCT could become a widely used tool in the management of LLD. This could potentially lead to better quality of life for many older adults, reducing the burden of depression and cognitive decline, and address the limitations and potential side effects associated with the use of antidepressants.

## Conclusion

This trial provides preliminary evidence of CCT improving mood, cognition, and BDNF level in LLD. With our populations ageing rapidly, LLD being prevalent, and older adults less likely to respond to antidepressants, CCT that targets executive dysfunction may serve as a safe and effective alternative or adjuvant treatment modality for LLD.

## Data availability statement

The raw data supporting the conclusions of this article will be made available by the authors, without undue reservation.

## Ethics statement

The studies involving humans were approved by Joint Clinical Research Ethics Committee of the Chinese University of Hong Kong and the New Territories East Cluster of the Hospital Authority. The studies were conducted in accordance with the local legislation and institutional requirements. The participants provided their written informed consent to participate in this study.

## Author contributions

SM: Conceptualization, Formal analysis, Investigation, Methodology, Project administration, Resources, Supervision, Writing – review & editing, Validation. KT: Data curation, Writing – review & editing. NL: Data curation, Writing – review & editing. CC: Data curation, Writing – review & editing. CL: Investigation, Software, Writing – review & editing. LL: Conceptualization, Methodology, Project administration, Resources, Supervision, Writing – review & editing. AL: Conceptualization, Formal analysis, Funding acquisition, Investigation, Methodology, Project administration, Resources, Supervision, Visualization, Writing – original draft, Writing – review & editing, Validation.

## Footnotes

^1^ www.randomization.com
