# Supplementary material for: Effect of computerized cognitive training on mood, cognition, and serum brain-derived neurotrophic factor level in late-life depression — a pilot randomized controlled trial
Source: Front Psychiatry. 2024 Jan 17;14:1287822. doi: 10.3389/fpsyt.2023.1287822 (PMC10827875; doi:10.3389/fpsyt.2023.1287822)
Supplement: Supplementary file 2 [file Data_Sheet_1.docx]

**Supplementary information**

**Structure of each session:**

1. Computerized cognitive training (experimental group):
2. Computer-based health educational program (control group):

**Different components of computerized cognitive training:**

1. Training on complex attention is based on computer-based attention training composed of stimulus cues and flanker task, in which the efficiency of different components of attention can be trained, with outcomes quantified by accuracy and reaction time (RT).
2. Training on executive control is based on the Internal Switch Training (IST) and the Balloon Analogue Risk Training (BART). IST targets on cognitive flexibility by training one’s ability to shift attention internally between alternative mental representations. BART, which is implicated in risk taking, trains one’s ability to balance the potential for reward and loss.
3. Training on working memory is based on the Picture Matching training.

*Complex Attention Training* Figure. 1. Schematic diagram of Complex Attention Training

*
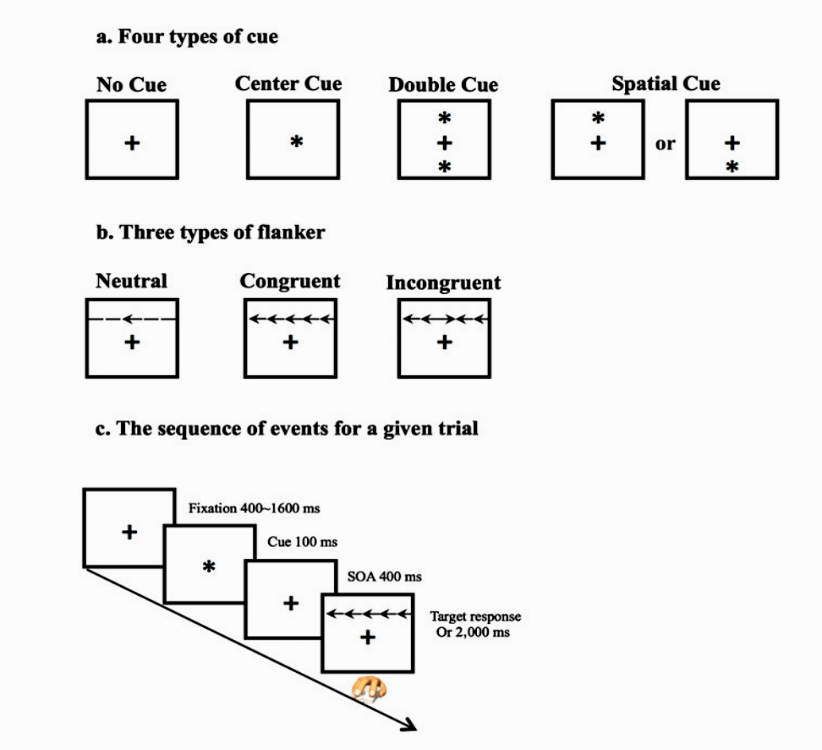
*The paradigm has 4 types of cue (no, center, double, and spatial cues; Figure 1a) and 3 types of flanker (neutral, congruent and incongruent; Figure 1b). In a given trial, a central cross-fixation point presents for 400–1600 milliseconds (ms) (random), followed by one of the 4 cues for 100 ms (Figure 1c). The target, which is an arrow at the centre with 2 flankers on each side, will then appear above or below the cross-fixation. The participant is instructed to indicate the direction of the target by pressing the right or left arrow button. Each trial lasts no more than 3 seconds, each block has 48 trials, and each session has 4 blocks. The attention task involves 3 components (alerting, orienting and executive control), and each outcome can be quantified by subtracting reaction time (RT) between one condition and the reference condition. The formulae of conventional scoring are:

- Efficiency of alerting = RTno cue – RTcenter cue
- Efficiency of orienting = RTcenter cue – RTspatial cue
- Efficiency of executive control = RTincongruent – RTcongruent

*Internal Switch Training (IST)*

This executive control training follows a block design, with 4 blocks in total and each block varying between 18 and 22 trials. In each block, a random number of pictures (18-22) is shown on the screen; participant has to press the button before the next picture is shown. He/she has to decide the category of each picture (e.g., food vs household) and make a silent count on the number of pictures he/she sees in each category. Participant reports his/her count at the end of the block (Figure 2).

Figure 2. Flow of the Internal Switching


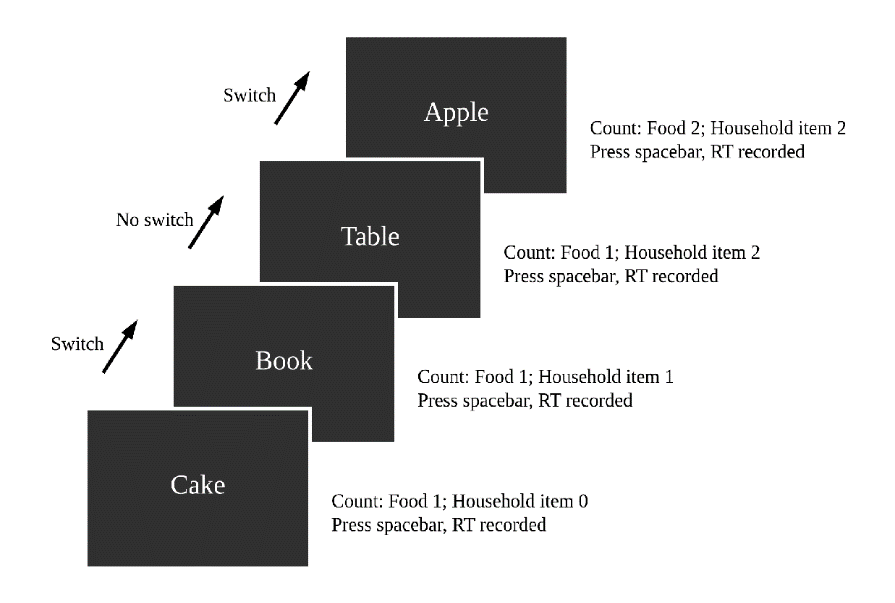
The delay between the presentation of picture and pressing of the button is recorded automatically as RT. Trials followed by a successive picture of the same category are known as the “no switch” trials; those followed by a picture from the other category represent the “switch” trials. The “Switching cost” is the extra RT needed to process the switch trials.

*Balloon Analogue Risk Training (BART)*


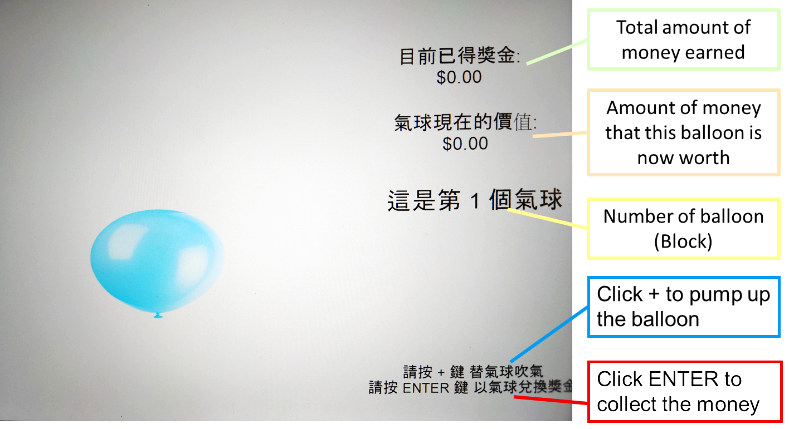
BART is a computerized training of risk-related constructs (impulsivity and sensation seeking) and risk-taking behaviours. In each block, a simulated balloon appears on the screen, and participant is asked to pump it up by pressing a button. Each press incrementally inflates the balloon until it explodes at a certain threshold. Participants are not informed about the threshold prior to each block; this facilitates testing their initial responses to the task and changes in response as they gain experience with the task contingencies. At any point before the balloon explodes, participant has the choice to end the trial and earn simulated money based on the size of the balloon, with larger size conferring more money, but no money will be earned for that block once the balloon explodes. As such, each pump is associated with both greater risk and greater potential reward. Each block starts with a new balloon and has 30 trials. Participant can see the amount of money potentially earned in the current block and the accumulated amount earned from previous blocks (Figure 3).

Figure 3. Screen capture of Balloon Analogue Risk Training

*Picture Matching*

In this visual working memory training, a series of pictures are shown on screen, one at a time, in which the participants are instructed to memorize them. Another series of pictures are then shown, which are a mix of old and new ones, and participants are asked to identify the ones that they have seen.
